# *CitAP2.10* activation of the terpene synthase *CsTPS1* is associated with the synthesis of (+)-valencene in ‘Newhall’ orange

**DOI:** 10.1093/jxb/erw189

**Published:** 2016-05-18

**Authors:** Shu-ling Shen, Xue-ren Yin, Bo Zhang, Xiu-lan Xie, Qian Jiang, Donald Grierson, Kun-song Chen

**Affiliations:** ^1^College of Agriculture & Biotechnology, Zhejiang University, Zijingang Campus, Hangzhou 310058, PR China; ^2^Zhejiang Provincial Key Laboratory of Horticultural Plant Integrative Biology, Zhejiang University, Zijingang Campus, Hangzhou 310058, PR China; ^3^The State Agriculture Ministry Laboratory of Horticultural Plant Growth, Development and Quality Improvement, Zhejiang University, Zijingang Campus, Hangzhou 310058, PR China; ^4^School of Biosciences, University of Nottingham, Sutton Bonington Campus, Loughborough, LE12 5RD, UK

**Keywords:** CitAP2/ERF, *Citrus sinensis*, citrus volatile, ethylene, sweet orange, terpene synthase, TPS, transcriptional regulation, valencene.

## Abstract

Aroma is a vital characteristic that determines the quality and commercial value of citrus fruits, and characteristic volatiles have been analyzed in different citrus species. In sweet orange, *Citrus sinensis*, the sesquiterpene (+)-valencene is a key volatile compound in the fruit peel. Valencene synthesis is catalyzed by the terpene synthase CsTPS1, but the transcriptional mechanisms controlling its gene expression are unknown. Here, the AP2/ERF (APETALA2/ethylene response factor) transcription factor, *CitAP2.10*, is characterized as a regulator of (+)-valencene synthesis. The expression pattern of *CitAP2.10* was positively correlated with (+)-valencene content and *CsTPS1* expression. Dual-luciferase assays indicated that *CitAP2.10* could trans-activate the *CsTPS1* promoter. Ethylene enhanced expression of *CitAP2.10* and this effect was abolished by the ethylene antagonist 1-methylcyclopropene. The role and function of *CitAP2.10* in (+)-valencene biosynthesis were confirmed using the Arabidopsis homolog (*AtWRI1*), which also transiently activated the *CsTPS1* promoter. Furthermore, transient over-expression of *CitAP2.10* triggered (+)-valencene biosynthesis in sweet orange fruit. These results indicate that *CitAP2.10* regulates (+)-valencene synthesis via induction of *CsTPS1* mRNA accumulation.

## Highlight

*CitAP2.10* is a novel AP2/ERF transcription factor associated with (+)-valencene content in sweet orange fruit and operates by modulating *CsTPS1* transcript abundance.

## Introduction

Over 1000 individual volatile organic compounds have been identified from plants (Qualley and Dudareva, 2009). These volatiles affect plant growth, development, and environmental adaptation (Kessler and Baldwin, 2001). For fruit, aroma is one of the most significant quality traits, potentially influencing consumer acceptance. Most fruit, such as mango (Singh and Saini, 2014), strawberry (Van de Poel *et al.*, 2014), pear (Moya-Leon *et al.*, 2006), melon (Gonda *et al.*, 2013), and citrus (Shi *et al.*, 2007) produce large amounts of volatiles. Fruits are particularly useful for aroma research, as specific compounds are unique to the fruit and are not found in other plant tissues, e.g., esters can be detected in mango fruit but are absent from leaves (da Silva *et al.*, 2012; Lalel *et al.*, 2003), and linalool and C10 lactones appear in ripe peach but are absent from leaves (Horvat and Chapman, 1990). Citrus fruit are also excellent materials for terpenoid research. The volatile terpenoids, a large and structurally diverse group of secondary metabolites, are represented by isoprenes (C5), monoterpenes (C10), and sesquiterpenes (C15). Volatile terpenoids are all derived from two C5-isoprene building units, isopentenyl diphosphate (IPP) and dimethylallyl diphosphate (DMAPP), which are synthesized from the mevalonate (MVA) and non-mevalonate (MEP) pathways (Mcgarvey and Croteau, 1995). Most of the terpenes are synthesized from their corresponding substrates by terpene synthases (TPS). In citrus species, several *TPS* genes have been characterized, including *CitMTSE1* (AB110636.1) from satsuma mandarin (*Citrus unshiu* Marcow, Shimada *et al.*, 2004), *RlemTPS1-3*(AB691531.1, AB266584.1, AB266585.1) from citrus jambhiri (Yamasaki and Akimitsu, 2007; Shishido *et al.*, 2012), and (+)-valencene biosynthesis-related *CsTPS1* (AF514289.1) from sweet orange (*Citrus sinensis*, Sharon-Asa *et al.*, 2003).

The mechanisms and identities of transcription factors regulating terpenoid biosynthesis have been characterized in model plants, where members of the ARF (auxin response factors), MYC (myelocytomatosis related proteins), MYB (myeloblastosis related proteins), and WRKY (amino-acid sequence WRKYGQK) families have been implicated. It has been reported that the Arabidopsis auxin responsive factors *AtARF6* and *AtARF8*, as well as *AtMYB21* and *AtMYB24*, can induce the production of sesquiterpenes (Mandaokar *et al.*, 2006; Reeves *et al.*, 2012), and *AtMYC2* can regulate synthesis of sesquiterpenes by binding to the promoter of *AtTPS11* and *AtTPS21* (Hong *et al.*, 2012). *PtMYB14* is involved in regulating the MVA pathway, as well as jasmonate metabolism, resulting in the release of volatile terpenoids in coniferous trees (Bedon *et al.*, 2010). Similarly, *NaWRKY3* and *NaWRKY6* participate in defense against herbivores in *Nicotiana attenuata* by producing terpenoids to attract predators and reduce the number of herbivorous larvae (Skibbe *et al.*, 2008). Despite the increasing number of investigations of the transcription factors regulating aroma in the plant kingdom, there have been few previous reports on the transcriptional regulation of fruit volatiles.

The formation of plant volatiles is regulated by both internal (developmental, tissue-specific) and external (environmental) factors. For instance, some volatile terpenoids can be induced or inhibited by environmental stresses such as wounding, insect and herbivore attack, and by hormones such as gibberellins and jasmonates (Faldt *et al.*, 2003; Feng *et al.*, 2008; Dicke *et al.*, 2009). Ethylene is an important hormone that regulates fruit ripening and quality, including aroma, and ethylene treatment induces ripening and volatile synthesis (Alexander and Grierson, 2002), while inhibition of ethylene production by antisense ACC (aminocyclopropanecarboxylate) oxidase RNA in melon prevents volatile production (Pech *et al.*, 2008, 2012). Ethylene response factors (*ERF*s) have recently been identified as targets for fruit ripening and quality research, and *SlERF*s have been demonstrated to be ripening-related transcription factors (Liu *et al.*, 2016); *MaERF*s have been shown to be involved in banana fruit ripening (Xiao *et al.*, 2013); *PsERF*s determine the rate of ripening in Japanese plum (El-Sharkawy *et al.*, 2009); apple *MdCBF* and kiwifruit *AdERF9* participate in fruit softening (Tacken *et al.*, 2010; Yin *et al.*, 2010); and persimmon *DkERF9/10/19/22* contributes to fruit deastringency (Min *et al.*, 2012, 2014). Although an increase in fruit aroma production is usually associated with fruit ripening, the potential role of *AP2/ERF* (APETALA2/ethylene response factor) transcription factors in fruit aroma has not previously been explored.

Using a citrus genome database, 126 *CitAP2/ERF* genes were isolated and most of them were found to be differentially expressed during citrus fruit development, which indicates their potential involvement in the ripening of citrus fruit (Xie *et al.*, 2014). In the present study, the transcriptional regulatory roles of *CitAP2.10*, a *CitAP2/ERF* family member, in regulating (+)-valencene production in citrus sweet orange ‘Newhall’ fruit were investigated during different stages of fruit development, and in response to treatment with ethylene and its action inhibitor 1-methylcyclopropene. A potential role for *CitAP2.10* in regulating citrus (+)-valencene production was examined by transient overexpression in citrus peels and the action on the promoter of the target gene *CsTPS1* was studied using a tobacco dual-luciferase assay.

## Materials and methods

### Plant materials and treatments

Fruit of eleven citrus cultivars from six species were collected at the commercial ripened stages (Table 1; related physiological data are indicated in Supplementary Table S1 at *JXB* online).

**Table 1.** Commercial ripened stages of 11 citrus cultivars

**Common name**	**Cultivar and species**	**Days after full bloom (DAFB**)
Bitter Orange	‘Goutoucheng’ (*Citrus aurantium*)	215
Citron	Lemon (*Citrus limon*)	210
	‘Bergamot’ (*Citrus medica*)	170
Hybrids	‘Huyou’ (*Citrus changshanensis*)	205
	‘Hongshigan’ (*Citrus reticulata× Citrus sinensis*)	215
Mandarin	Satsuma (*Citrus unshiu*)	180
	‘Ponkan’ (*Citrus reticulata*)	225
Pummelo	‘Yuhuan’ (*Citrus grandis*)	210
	‘Zaoxiang’ (*Citrus grandis*)	200
Sweet Orange	‘Newhall’ (*Citrus sinensis*)	210
	‘Fengjie’ (*Citrus sinensis*)	210

Fruits of the sweet orange (*Citrus sinensis*) cultivar ‘Newhall’ were obtained from a commercial orchard in Songyang (Zhejiang, China) at seven different developmental stages, namely at 120, 135, 150, 165, 180, 195 and 210 d after full bloom (DAFB) in 2011. In order to study the effect of ethylene on the biosynthesis of citrus sesquiterpenes, ‘Newhall’ sweet orange fruit were collected at 150 DAFB from the same orchard in 2012, treated separately with 40 μl l^−1^ ethylene or 1 μl l^−1^ 1-methylcyclopropene (1-MCP) and stored at 20 °C for 5 d after harvest. For treatments, the harvested fruit were randomly divided into three groups for each treatment, as three biological replicates. Peel samples taken from the equatorial portion of fruits were cut into small pieces, immediately frozen in liquid nitrogen and stored at –80 °C for subsequent study.

### Volatile compounds analysis

The frozen tissues were ground in liquid nitrogen and 1g was weighed into a 15-ml headspace vial containing 5ml saturated sodium chloride solution. Before capping of the vial, 50 μl of 1-Hexanol (0.1%, v/v) was added as an internal standard. After vigorous vortexing, the samples were incubated at 40 °C for 30min with continuous agitation (600rpm). Following this, a SPME fiber coated with 50/30 μm divinylbenzene/carboxen/polydimethylsiloxane (DVB/CAR/PDMS) (Supelco Co., Bellefonte, PA, USA) was used to extract the volatiles under the same conditions (40 °C, 600rpm). Volatile analysis was carried out with an Agilent 7890A gas chromatograph (GC) coupled to an Agilent 5975C Network Mass Selective Detector (MS, inert XL MSD with triple-axis detector). After extraction, the fiber was exposed to the GC injection port at 250 °C for 5min in splitless mode. Samples were separated using a HP-5MS column (5% phenyl methyl siloxane, 30 m × 0.25mm × 0.25 μm, J&W Scientific, Folsom, CA, USA). Helium was used as a carrier gas at 1.0ml min^−1^. For the peel samples, the oven temperature was programmed to start at 40 °C for 3min, and then ramped to 70 °C at a rate of 3 °C min^−1^, followed by a second ramp to 130 °C at a rate of 1 °C min^−1^, and a third ramp to 230 °C at a rate of 15 °C min^−1^. MS conditions were as follows: ion source, 230 °C; electron energy, 70eV; GC-MS interface zone, 250 °C, and a scan range of 35–350 mass units. Volatiles were identified based on the database of the NIST/EPA/NIH Mass Spectral Library (http://chemdata.nist.gov/) and the Wiley Registry of Mass Spectral Data (http://onlinelibrary.wiley.com/book/10.1002/9780470175217). The identities of most of the volatiles were then confirmed by comparison with authentic standards. The internal standards were used for compensating for differences between samples, and the abundance of each volatile was calculated as its peak area. The identification of (+)-valencene was made by comparison with the mass spectrum of the standard compound, retention time, and its retention index. The results are also displayed as a heatmap in order to provide an overview of differences in volatile compounds among the citrus fruit samples. ANOVA was used for identification of characteristic volatiles. The differences indicated in Supplementary Table S4 are based on Tukey’s test at the 5% level (SAS version 8.0, SAS Institute, Cary, NC, USA).

### RNA extraction and cDNA synthesis

Total RNA was extracted from frozen tissues (0.3g) of citrus peel according to the CTAB (cetyltri-methylammonium bromide) method developed from Chang *et al.* (1993). After removing the gDNA contamination by TURBO DNase (Ambion), 1.0 μg DNA-free RNA was used to synthesize the cDNA by means of an iScript cDNA Synthesis Kit (Bio-Rad). RNA extractions were carried out in parallel on three biological treatment replicates and samplings.

### Gene isolation and sequence analysis

*CitAP2/ERF* genes and the promoter of *CsTPS1* were isolated based on the sequences in the online citrus genome database (http://citrus.hzau.edu.cn/, *Cs5g12900*), and full-length *AtWRIs* (Arabidopsis AP2/ERF) genes were obtained based on the sequences in TAIR (The Arabidopsis Information Resource, https://www.arabidopsis.org/) with the primers listed in Supplementary Table S2. The gene sequences were translated by Primer Premier 5.0. Alignment and phylogenetic analysis was carried out by ClustalX (V.1.81) and Mega 4.0.2.

### Real-time quantitative PCR

The oligonucleotide primers of *CitAP2/ERF* genes used for real-time quantitative PCR were as described in Xie *et al.* (2014). The *CsTPS1* primers were designed by primer3 (http://frodo.wi.mit.edu/primer3) and are described in Supplementary Table S3. The specificity of the primers was tested with melting curves and resequencing of PCR products.

The real-time PCR was carried out with a Ssofast Eva Green Supermix Kit using a CFX96 instrument (Bio-Rad). The PCR mixture and reactions were as described in our previous report (Yin *et al.*, 2012). Abundance of cDNA templates was monitored with citrus β-actin (Pillitteri *et al.*, 2004). △△Ct was used to calculate the relative expression levels of genes.

### Dual-luciferase assay

Transactivation activities of AP2/ERF on the target promoter were measured with dual-luciferase assays according to previous our reports (Yin *et al.*, 2010; Xu *et al.*, 2014).

The full-length *CitAP2.10* and *AtWRI1-4* (the homolog of *CitAP2.10* in Arabidopsis) sequences were amplified with the primers described in Supplementary Table S2 and were inserted into a pGreenII 0029 62-SK vector. The promoter of *CsTPS1* (573bp) was constructed in the pGreenII 0800-LUC vector. All constructs were individually electroporated into *Agrobacterium tumefaciens* GV3101 and stored as glycerol stock at –80 °C. *Agrobacterium* cultures were prepared with infiltration buffer (10mM MES, 10mM MgCl_2_, 150mM acetosyringone, pH 5.6) to an OD_600_ of 0.75. The mixtures of transcription factors 1ml) and promoters (100 μl) were infiltrated into tobacco leaves by needleless syringes. Tobacco plants were grown in a growth chamber with a light/dark cycle of 16 : 8h, at 24 °C.

Four-week-old plants were prepared for injection. Enzyme activities of firefly luciferase and renilla luciferase were assayed using dual-luciferase assay reagents (Promega), at 3 d after infiltration. For each transcription factor–promoter interaction, at least three independent experiments were performed, with four replicates in each experiment.

### Transient overexpression in citrus peel

In order to determine the role of the *CitAP2.10* gene in the regulation of (+)-valencene synthesis in citrus, transient overexpression analysis was performed on ‘Newhall’ fruit peel, as used previously for various other fruits, such as apple (Li *et al.*, 2012), persimmon (Min *et al.*, 2012), and strawberry (Hoffmann *et al.*, 2006; Salvatierra *et al.*, 2013). The *Agrobacterium* cultures carrying empty vector (SK) and constructs containing the target gene (*CitAP2.10*) were infiltrated into the same fruit, on opposite sides of the equatorial portion. Five days after infiltration, the peel near (<12mm) the infiltration point (without including the infiltration spot) was collected and immediately frozen in liquid nitrogen. These samples were stored at –80 °C for analysis of volatiles (1g for volatile measurements for each replicate) and endogenous *CsTPS1* expression (0.3g for RNA extractions for each replicate).

### Statistical analysis

The statistical significance of differences was calculated by single-factor ANOVA using Microsoft Excel (2013 version). Least-significant difference (LSD) at the 5% level was calculated using DPS7.05 (Zhejiang University, Hangzhou, China). Figures were drawn using Origin 8.0 (Microcal Software Inc.).

## Results

### (+)-Valencene is one of the characteristic volatile compounds in mature sweet orange

**Fig. 1.**
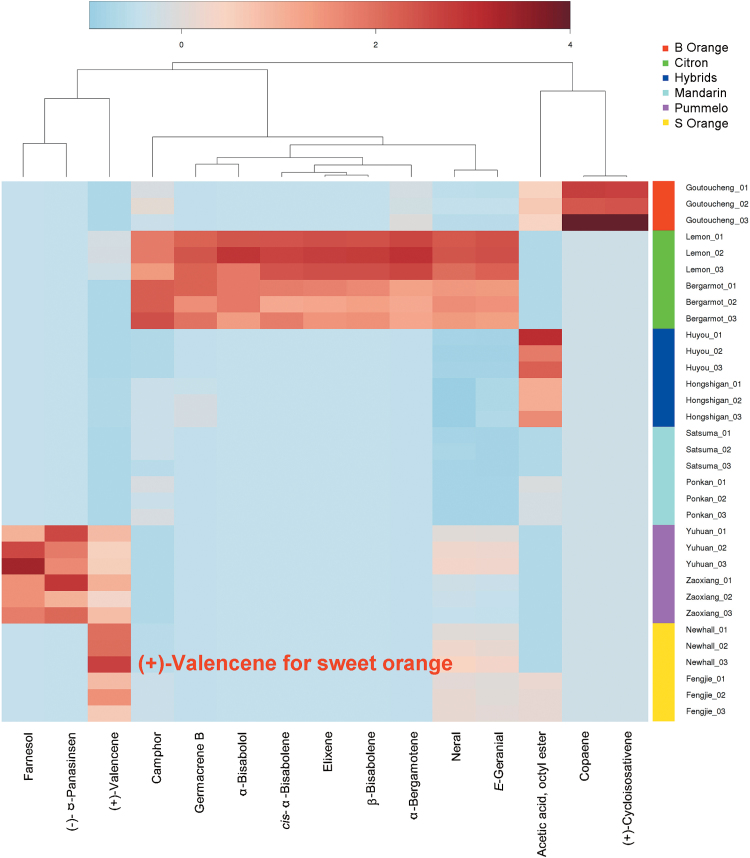
Heatmap generated from hierarchical cluster analysis (HCA) based on intensity patterns of volatiles in the fruit peel of six species of citrus. Abundance of the volatile compounds, evaluated by HS SPME GC-MS from three biological replicates, is represented by colors on the *X*-axis (top). The six citrus species and the corresponding fruit samples are on the *Y*-axis (right).

Following earlier comparative studies (Del Rio *et al.*, 1992; González-Mas *et al.*, 2011; Miyazaki *et al.*, 2011), the volatile compounds from six species of citrus fruit (mandarin, sweet orange, bitter orange, hybrids, pummelo, and citron) plus some new cultivars, (satsuma, ‘Newhall’ and ‘Fengjie’) were analysed. A total of 119 volatiles were identified from these citrus fruit, including 16 monoterpenes, 37 sesquiterpenes, 36 monoterpenoids, 10 sesquiterpenoids, 14 aliphatic aldehydes and alcohols, and six aliphatic esters (Supplementary Table S4). Differences in volatile profiles were observed between the samples and fifteen characteristic volatile compounds from citrus species were successfully identified (Fig. 1). Bitter orange was characterized by (+)-cycloisosativene and copaene. The volatile profile of citron fruit was complex: *cis*-α-bisabolene, α-bisabolol, elixene, and β-bisabolene were exclusively found in citron but germacrene B, camphor, α-bergamotene, (*E*)-geranial, and neral were also found to be characteristic for citron due to their relative higher concentrations as compared to the other species. Hybrid cultivars had a higher concentration of octylacetate while the pummelo species were characterized by (–)-α-panasinsen, farnesol and (+)-valencene. Sweet orange was characterized by the volatile compound (+)-valencene, which was much more abundant than in other citrus species (Fig. 1).

### Screening for *CitAP2/ERF* genes encoding proteins that activate the *Terpene Synthase 1* (*CsTPS1*) promoter

**Fig. 2.**
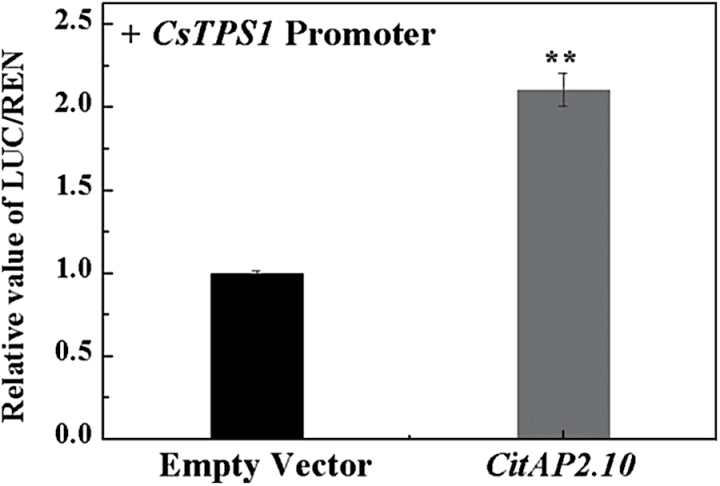
*In vivo* interaction between the *CitAP2.10* protein and the promoter of *CsTPS1*. Samples were infiltrated into *Nicotiana benthamiana* leaves. For the transcription factor–promoter interactions, three independent experiments were performed (four replicates in each experiment). Firefly luciferase (LUC) and renilla (REN) luciferase were assayed 3 d after infiltration. The ratio of LUC/REN to the empty vector plus promoter was used as a calibrator (set as 1). Error bars indicate SE from four biological replicates (**P<0.01).

*CsTPS1* has previously been characterized as the key gene encoding the enzyme terpene synthase that catalyzes (+)-valencene biosynthesis (Sharon-Asa *et al.*, 2003). Therefore, we screened a large set of citrus transcription factors for activation or inhibition using the promoter of *CsTPS1* and a dual-luciferase assay (Supplementary Fig. S1). Previously, an AP2 domain transcription factor (*CrORCA3*) was reported as a regulator of terpenoid indole alkaloids (van der Fits and Memelink, 2000). However, a linkage between a *CitAP2/ERF* transcription factor and developmental accumulation of (+)-valencene in sweet orange fruit has not yet been demonstrated. Utilizing the promoter of *CsTPS1* and a dual-luciferase assay, the *CitAP2/ERF* genes were screened to identify any that activated the *CsTPS1* promoter (–573~–1bp region). Among the *CitAP2/ERFs*, one transcription factor, *CitAP2.10*, showed a 2.1-fold trans-activation effect on the *CsTPS1* promoter (Fig. 2).

### Association of *CitAP2.10* and *CsTPS1* expression and (+)-valencene accumulation during ‘Newhall’ fruit development

**Fig. 3.**
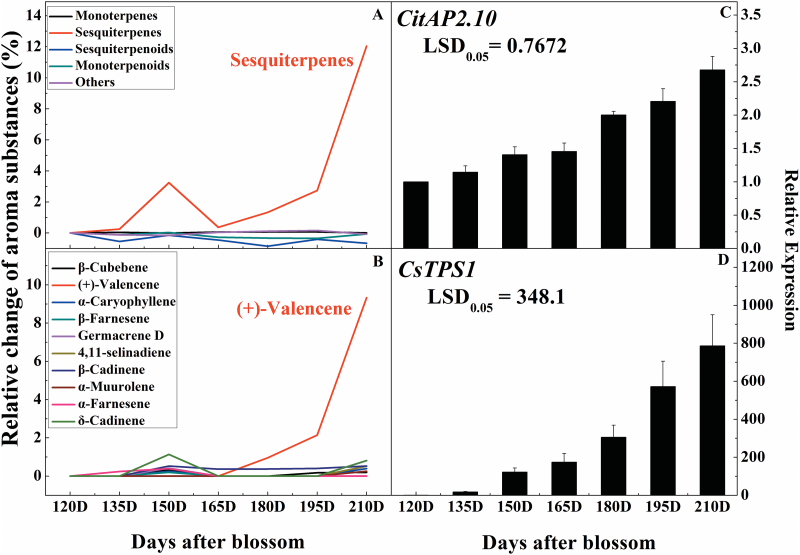
(A), (B) Comparison of the relative change in aroma substances during fruit development. The relative changes in aroma substances were calculated using the percentage content and compared to the aroma of fruits 120 d after blossom. (C), (D) *CitAP2.10* and sesquiterpene synthase (*CsTPS1*) gene expression patterns at different fruit developmental stages. The transcriptional analysis of ethylene-responsive factor *CitAP2.10* and sesquiterpene synthase *CsTPS1* was conducted by real-time PCR. Gene expression was calculated relative to the fruits 120 d after blossom (set as 1). Error bars indicate ±SE from three biological replicates. The values of LSD represent the least-significant differences at *P*=0.05.

The percentage content of five classes of aroma substances in citrus peel (monoterpenes, sesquiterpenes, sesquiterpenoids, monoterpenoids, others) was analysed and the relative change in content during fruit development was measured, using fruits harvested 120 DAFB as a reference point (set as 0). The most significant class of volatiles that accumulated was the sesquiterpenes (Fig. 3), which had doubled by 180 DAFB and increased 12-fold by 210 DAFB, while the other four classes of aroma substances remained relatively less abundant (Fig. 3). The main sesquiterpene that accumulated during fruit development was (+)-valencene, which increased by 9-fold and 93-fold at 180 DAFB and 210 DAFB, respectively, which was much higher than any of the other volatiles (Fig. 3).

The expressions of *CitAP2.10* and *CsTPS1* were analysed in parallel with volatile accumulation in sweet orange ‘Newhall’ (Fig. 3). The results indicated that mRNAs for *CsTPS1* and *CitAP2.10* accumulated during maturation of sweet orange fruit (120 DAFB to 210 DAFB). Thus, *CitAP2.10* expression was positively correlated with *CsTPS1* activity and (+)-valencene accumulation.

### Modulation by ethylene and 1-MCP of (+)-valencene content and expression of *CitAP2.10* and *CsTPS1*

**Fig. 4.**
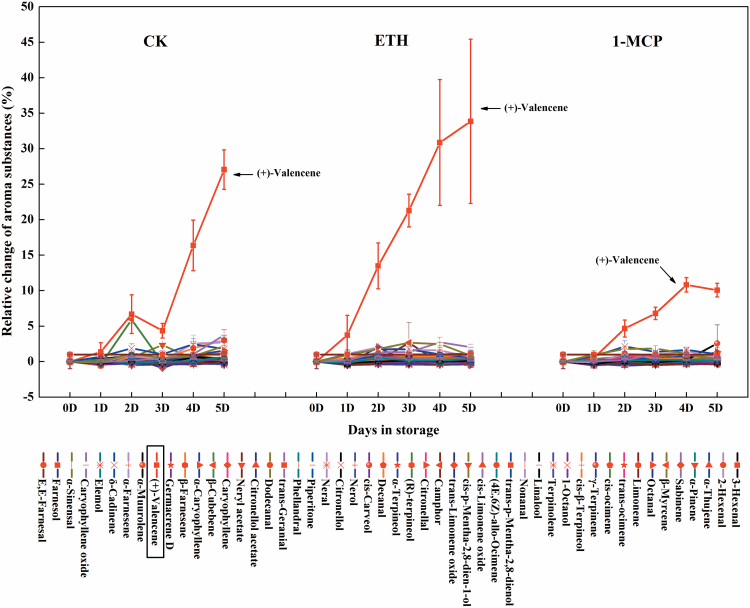
Effects of ethylene and 1-MCP on production of aroma substances by fruit of sweet orange ‘Newhall’. Fruits at 150 d after blossom were treated with ethylene (ETH, 40 μl l^−1^, 12h), 1-MCP (1 μl l^−1^, 12h), or air (CK, the control) separately and stored at 20 °C for 5 d. The quantity of aroma substances in treated fruits was calculated using the percentage content and compared to fruits at day 0 (fruits 120 d after full bloom). Error bars indicate SE from three replicates.

**Fig. 5.**
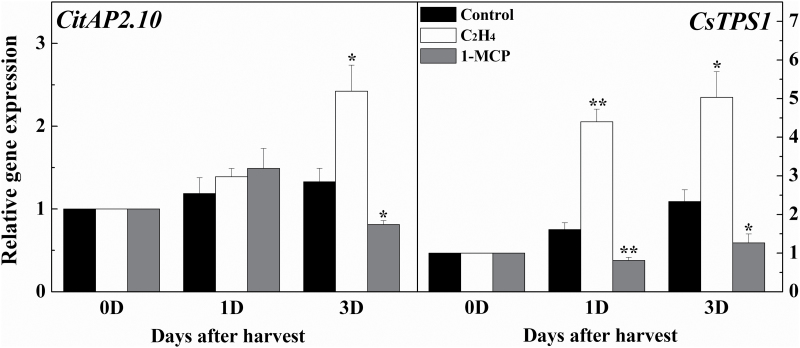
Transcriptional analysis of *CitAP2.10* and sesquiterpene synthase *CsTPS1*. Transcripts of *CitAP2.10* and *CsTPS1* were measured by real-time PCR in response to ethylene (C_2_H_4_) and 1-MCP treatment and compared to the control (CK). The fruits were treated 150 d after blossom with ethylene (40 μl l^−1^, 12h), 1-MCP (1 μl l^−1^, 12h) or air (CK, the control) separately and stored at 20 °C. Relative expressions were caluclated with respect to fruits at 0 d after harvest (Control 0 d, set as 1). Error bars indicate SE from three biological replicates (*P<0.05; **P<0.01).

The correlation between *CitAP2.10* and (+)-valencene was further tested by treatment with exogenous ethylene and 1-MCP (an ethylene antagonist). The results indicated that ethylene enhanced and 1-MCP inhibited (+)-valencene biosynthesis. In control fruit (Fig. 4) (+)-valencene content increased by approximately 16.4% between 0 d and 4 d, whereas treatment with ethylene enhanced valencene content by 30.9% and treatment with 1-MCP reduced it to 6.89% over the same time period. Further analysis of gene expression indicated that *CsTPS1* mRNA showed the same accumulation pattern as (+)-valencene in fruit in response to both ethylene and 1-MCP treatment. *CitAP2.10* transcript abundance was significantly higher than in the control following ethylene treatment, and significantly lower in fruit treated with 1-MCP relative to the control (Fig. 5).

### *Trans*-activation activity of *AtWRI1*, a homolog of *CitAP2.10* on the *CsTPS1* promoter

**Fig. 6.**
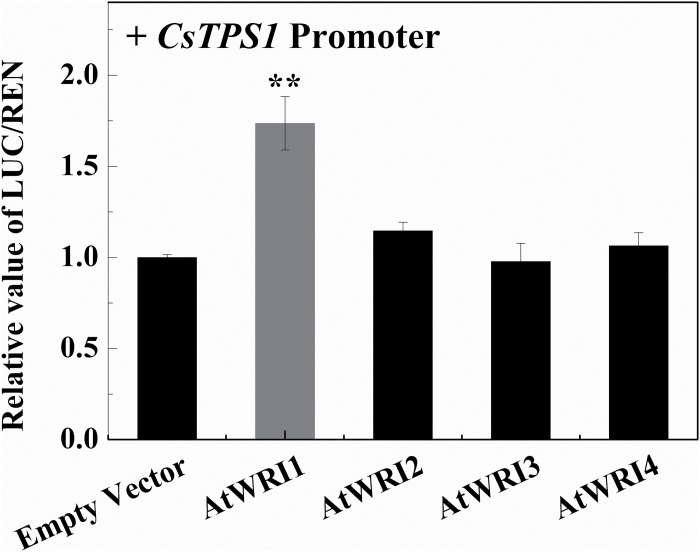
Analysis of the *in vivo* interaction between four different *AtWRI*s and the *CsTPS1* promoter using the dual-luciferase assay. Error bars indicate SE from four biological replicates (**P<0.01).

The results above indicate that *CitAP2.10* is positively associated with *CsTPS1* and also with the accumulation of the metabolite (+)-valencene. In order to verify the regulatory role of CitAP2/ERF, four Arabidopsis *AtWRI* genes showing the closest similarity to *CitAP2.10* (Supplementary Fig. S2) were isolated and their *trans*-activation activities were tested using the dual-luciferase assays. The results (Fig. 6) indicated that *AtWRI1* functioned in a similar way to *CitAP2.10* and was able to activate the *CsTPS1* promoter, while the other three *AtWRI* genes had limited effects.

### Transient over-expression in ‘Newhall’ fruits

**Fig. 7.**
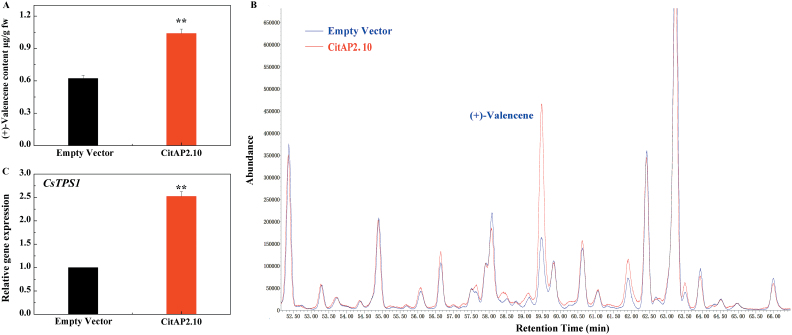
Transient expression of *CitAP2.10* in ‘Newhall’ citrus peel. (A) (+)-Valencene content in peel infiltrated with *CitAP2.10*, driven by the CaMV 35S promoter. Error bars indicate SE from three biological replicates (**P<0.01). (B) Volatiles emitted from peel injected with Empty Vector (blue) and *CitAP2.10* (red). (C) Relative *CsTPS1* gene expression in citrus peel injected with Empty Vector and *CitAP2.10*. Error bars indicate SE from three biological replicates (**P<0.01).

Citrus is a perennial woody plant and stable transformation is difficult and time-consuming. Thus, a more rapid and efficient transient overexpression assay was chosen to examine the function of *CitAP2.10 in vivo*. *CitAP2.10* and the empty vector were separately injected on opposite sides of the equatorial portion in the same fruit and the volatiles of these parts were analysed 5 d after infiltration. The results indicated that introducing *CitAP2.10* accelerated (+)-valencene synthesis (Fig. 7). The (+)-valencene content of the peels infiltrated with *CitAP2.10* was 1.04 μg.g^−1^, representing a significant (*P*<0.01) increase compared with peels infiltrated with empty vector (0.62 μg.g^−1^). Accumulation of endogenous *CsTPS1* mRNA occurred concomitantly with the increase in (+)-valencene (Fig. 7).

## Discussion

### (+)-Valencene is a characteristic terpene component in sweet orange

Aroma is a vital index of fruit quality, especially for citrus. Six citrus species covering eleven cultivars were examined, including some cultivars native to China, such as ‘Huyou’, ‘Goutoucheng’, and ‘Bergamot’. The results showed that volatiles were distributed differently in the various citrus samples. For mandarin, D-limonene, linalool, γ-terpinene, β-myrcene, α-pinene, (R)-(+)-citronellal, (-)-perillaldehyde, and (*E*, *E*)-2,4-decadienal were found to be abundant, which was similar to previous reports (Minh Tu *et al.*, 2002; Njoroge *et al.*, 2005; Miyazawa *et al.*, 2010). For citron fruit, apart from (*E*)-geranial and neral that have been identified previously (Venturini *et al.*, 2010), the characteristic volatile compounds consisted of germacrene B, (*Z*)-α-bisabolene, α-bisabolol, elixene, β-bisabolene, camphor, and α-bergamotene (Supplementary Table S4). In addition, some volatiles were unique to certain citrus fruit. Elixene was not detected in any of the species except those of citron, for which it is a unique volatile. In agreement with the report by Minh Tu *et al.* (2002), the detected peak area of δ-elemene was more than 0.005% but less than 0.05% in sweet orange and citron (Supplementary Table S4).

Valencene contributes to the powerful citrus aroma and is a characteristic component of ‘Sanguinello’ and ‘Moro’ juice (*Citrus sinensis*) (Maccarone *et al.*, 1998). It traditionally acts as a marker due to the statistical correlation with the oil quality of citrus peel (Elston *et al.*, 2005). We found that different species of citrus fruit had a great divergence in (+)-valencene content. The characteristic volatile compound in sweet orange, (+)-valencene, was much more abundant than in other citrus species (Fig. 1), which is consistent with the results of Maccarone *et al.* (1998). Based on a previous report (Sharon-Asa *et al.*, 2003) and our results, (+)-valencene accumulation could also be considered as a characteristic for ripening sweet orange, as indicated by the burst of (+)-valencene production during late developmental stages (Fig. 3). Studies on citrus genomics indicate that sweet orange may be derived from pummelo (Xu *et al.*, 2013), which is consistent with the detection of (+)-valencene in two pummelo cultivars (‘Yuhuan’ and ‘Zaoxiang’, Fig. 1).

### Ethylene and 1-MCP modulated (+)-valencene biosynthesis in sweet orange fruit

Physiological analysis indicated that (+)-valencene is not only influenced by developmental stage, but also by stimuli such as the plant hormone ethylene. Ethylene regulation of the synthesis of volatile compounds has been widely studied in various fruits, including ‘Charentais’ melons (Flores *et al.*, 2002), apple (Defilippi *et al.*, 2005), and kiwifruit (Atkinson *et al.*, 2011). The results of the current study demonstrated that ethylene enhanced (+)-valencene emission, which is similar to the findings of Sharon-Asa *et al.* (2003), whereas 1-MCP, an ethylene action inhibitor, reduced (+)-valencene emission in ‘Newhall’ fruit. Citrus is considered a non-climacteric fruit, yet according to a previous study, they are able to respond to ethylene (Katz *et al.*, 2004). The results of ethylene treatment and especially 1-MCP treatment lead to the conclusion that activation of the ethylene signaling pathway in citrus stimulates (+)-valencene synthesis.

### *CitAP2.10* modulates *CsTPS1* transcription

The impact of ethylene and 1-MCP on (+)-valencene synthesis led us to investigate the role of *CitAP2/ERF* genes in transactivation of the *CsTPS1* promoter. The correlation between gene expression and (+)-valencene synthesis together with the dual-luciferase assays all support the conclusion that *CitAP2.10* is involved in the regulation of (+)-valencene synthesis via *CsTPS1*. Previously, some *AP2/ERF* transcription factors have also been shown to be involved in the regulation of terpenoid synthesis in non-fruit systems. For example, *CrORCA3* (an AP2 domain transcription factor) overexpression resulted in increased accumulation of terpenoid indole alkaloids in *Catharanthus roseus* MP183L cultures (van der Fits and Memelink, 2000), while *AaERF1* and *AaERF2* induced artemisinin synthesis by binding to the promoter of *AaADS* directly (Yu *et al.*, 2012). However, none of these genes were reported to be involved in (+)-valencene biosynthesis. Thus, the results of the current study identify *CitAP2.10* as a new regulator of terpenoid biosynthesis, and also as the first transcription factor involved in (+)-valencene production. However, the regulatory mechanisms of *CitAP2.10* still require further investigation.

### Functional characterization of *CitAP2.10* in (+)-valencene regulation

Citrus is a perennial tree that is difficult to stably transform. Thus, transient overexpression experiments were conducted to test the role of *CitAP2.10*. Sweet orange peel infiltrated with *CitAP2.10* exhibited a significant increase in the level of (+)-valencene. In parallel, *CsTPS1* mRNA increased very significantly in response to *CitAP2.10*. Although further functional analysis needs to be performed with a stable transformation system, all the current results strongly indicate that *CitAP2.10* can up-regulate synthesis of (+)-valencene via modulating transcription of *CsTPS1*. Recently, using a similar approach in tobacco leaves, apple *MdoOMT1* and kiwifruit *NAC* and *EIL* transcription factors were shown to participate in biosynthesis of methylated phenylpropenes and terpenes, respectively (Nieuwenhuizen *et al.*, 2015; Yauk *et al.*, 2015), but the involvement of *AP2/ERF* members in fruit terpene synthesis has not been previously reported. The identification of *CitAP2.10* means it could be used as target for genetic modification for manipulation of (+)-valencene content to meet marketing requirements.

### Activity of *CitAP2.10*-related genes in Arabidopsis

In order to investigate the conservation of this function across species, a further experiment was conducted to isolate *CitAP2.10* homologs from Arabidopsis (*AtWRI1-4*). *CitAP2.10* has a tandem repeat of two AP2 domains and belongs to the AP2 subfamily, based on data from phylogenetic analysis (Licausi *et al.*, 2013). *AtWRI*s also belong to the AP2 subfamily (To *et al.*, 2012). However, the possible relationship between *AtWRI*s and terpenoids has not yet been reported. In Arabidopsis, *AtWRI*s have been reported to be responsible for modulating the rate of acyl chain production and fatty acid synthesis (To *et al.*, 2012). Here, of the four *AtWRI*s, *AtWRI1* was shown to interact with the *CsTPS1* promoter, producing a response of similar magnitude to the effect of *CitAP2.10*. These results suggest that *AtWRI1* might have conserved functions in regulating the production of terpenoids in Arabidopsis.

**Fig. 8.**
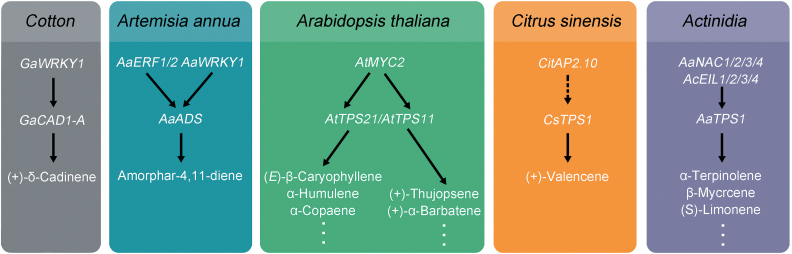
Transcription factors that regulate *TPS* and biosynthesis of sesquiterpenes in different species. The information was obtained from Xu *et al.*, 2004 (cotton); Yu *et al.*, 2012 (*Artemisia*); Hong *et al.*, 2012 (*Arabidopsis*); and Nieuwenhuizen *et al.*, 2015 (*Actinidia*).

In conclusion, *CitAP2.10* is a novel AP2/ERF transcription factor that is associated with (+)-valencene content in sweet orange fruit by modulating *CsTPS1* transcript abundance. The Arabidopsis transcription factor, *AtWRI1*, a *CitAP2.10* homolog previously described as a key regulator of fatty acids (To *et al.*, 2012), could also *trans*-activate the *CsTPS1* promoter, indicating that the function of *CitAP2/ERF* in (+)-valencene regulation may be conserved in various plants (Fig. 8). This result suggests an additional function for Arabidopsis *AP2/ERF* members. Despite the significant *in vivo* effects of *CitAP2.10* on *CsTPS1* and (+)-valencene content, the underlying regulatory mechanisms will require further investigation.

## Supplementary Data

Supplementary data are available at *JXB* online.

Table S1. Physiological data for mature citrus fruit from 11 commercial cultivars.

Table S2. Primers for full-length sequence amplification and promoter isolation.

Table S3. Primers for real-time PCR.

Table S4. Contents of 119 volatile compounds in citrus fruit from different species.

Figure S1. *In vivo* interaction of ethylene responsive factors and the promoter of *CsTPS1*.

Figure S2. Phylogenetic analysis of *CitAP2.10* and Arabidopsis *AP2* genes.

Supplementary Data
