# Supplementary material for: CitAP2.10 activation of the terpene synthase CsTPS1 is associated with the synthesis of (+)-valencene in ‘Newhall’ orange
Source: J Exp Bot. 2016 May 18;67(14):4105–15. doi: 10.1093/jxb/erw189 (PMC5301923; doi:10.1093/jxb/erw189)
Supplement: Supplementary Data [file supp_67_14_4105__index.html]

 CitAP2.10 activation of the terpene synthase CsTPS1 is associated with the synthesis of (+)-valencene in ‘Newhall’ orange — CitAP2.10 activation of the terpene synthase CsTPS1 is associated with the synthesis of (+)-valencene in ‘Newhall’ orange — Supplementary Data 

# *CitAP2.10* activation of the terpene synthase *CsTPS1* is associated with the synthesis of (+)-valencene in ‘Newhall’ orange

## Supplementary Data

Data files

- supplementary\_tables\_S1\_S4\_figures\_S1\_S2.pdf - Supplementary Data
