# Supplementary material for: CitAP2.10 activation of the terpene synthase CsTPS1 is associated with the synthesis of (+)-valencene in ‘Newhall’ orange
Source: J Exp Bot. 2016 May 18;67(14):4105–15. doi: 10.1093/jxb/erw189 (PMC5301923; doi:10.1093/jxb/erw189)
Supplement: Supplementary Data [file supp_erw189_supplementary_tables_S1_S4_figures_S1_S2.pdf]

Table S1. 11 citrus cultivar fruit color and taste at commercial ripened stages

Species	Cultivars	CCI*	TSS/TA[&]
Bitter Orange	Goutoucheng	1.78±0.06	1.36
Citron	Lemon	0.23±0.04	1.42
	Bergamot	2.17±0.13	—
Hybrids	Huyou	2.35±0.03	11.34
	Hongshigan	10.61±0.08	12.04
Mandarin	Satsuma	7.08±0.05	15.85
	Ponkan	7.13±0.14	15.81
Pummelo	Yuhuan	0.62±0.02	13.14
	Zaoxiang	0.01±0.01	12.46
Sweet Orange	Newhall	9.34±0.20	13.37
	Fengjie	8.56±0.21	12.58

*CCI: Citrus color index

[&] TSS, Total soluble solids; TA, Titratable acid

Table S2. Primers for full-length amplification and promoter isolation

Gene name	Forward primers (5’ to 3’)	Reverse primers (3’ to 5’)
CitAP2.10	ATGGCGAAAACCTCAAGGCA	GGCCTAATTATCAAGTTCAC
CitAP2.13	ATGATGGCGTCTTCTTCATCGG	TCATTCCAAGTGCAGCTCCAAG
CitAP2.14	CGCGGTGGCGGCCGCATGTTGGATCTCAACTTGAGTCTG	GGGGGATCCACTAGTCTTAGGGTACAAGCACATTCCTTT
CitRAV1	CCTCTTGTAGACACACATAACACACATG	GATGTTACAAAGCTCCAATC
CitSoloist1	GCAATATTTGGATCCATCATGGTGAG	TCTAAGAGGCTGATGAGTCTTGCCC
CitERF2	ATGGCAGCAGCTATAGATATG	GCGCTTAAAACTCAGATAATTTTC
CitERF4	ATGGAAGGATTTCAAGAAAATA	CTAATTGGCAAGAACTTCCCAA
CitERF7	CGCGGTGGCGGCCGCGCGTATTCATCATCAAGTGTATGG	GGGGGATCCACTAGTAGCGGAGCAGAGCTAGTTTAATTT
CitERF8	ATGGTGAGGCCGAGCGGC	TCAAAAGGTCCACAGACCAG
CitERF9	ATGGTGAAGGCTGAGCAAAGG	TCAGCAGAAACTCCATAAACGAA
CitERF14	CCACAACATTATATAATGACTGAGTGG	GAATTAGTGTTGCCACAACAGAGACTC
CitERF15	GCGGTGGTTGTCAGTTGTGATGGCAA	CTACTGATACTCCCACAAG
CitERF16	ATGGACATATTATTCGGCCAAG	TCAAATTGAATGACTCCAC
CitERF17	ATGAGTGAACCGCAAAACTCATC	TCAGGAATCCCACAACAAACCTT
CitERF18	ATGGCTGATCCTCATCAAAGAG	TTAAGTGTAGCTCCATAAACTT
CitERF19	ATGGATTCTCTATCAAGCTG	TTAAACAGAATAACTCCATAAGG
CitERF21	GGCCCTAAAAATACCACCATGC	ATTAAGGGAATTTCCAAAGG
CitERF23	GTAAATGGAGCAACAACCATACAC	CAATCTACAAACATGCCATGGACGGCGGCT
CitERF24	CTAATTCTACTGCATGGATGC	CCTAAGGGTCCCAAAGGGAGTCGCTT
CitERF27	GAGCTCATGGCCGCTGCCCCGTCAA	GGGCCCTCAAAAGTAGCTCCACAAA
CitERF28	TCCCTTTGTCTCGTTAATGG	TTAGTCACTCCACAAAGTAAAC
CitERF30	CATTACACGACTCCATGGAAAC	TTAATCCCATATGGACTCAACCTGC
CitERF31	ATGGCTATTCAAAGCAAAGATT	TCAAAATCCAAAATCTGATTCC

CitERF32	ATGGAGAGTTACAAAAAATC	TCAGAAGTTCCAAATGGAGG
CitERF33	ATGTCAAAAGCACTTATGGGTGG	TCAAACGCACCACGGAGT
CitERF39	ATGGTACAATCAAAGAAGTTCAGAGG	TTAGATCTCACTGGAGGGAGAAG
CitERF40	ATGGCCAGACCACAGCAG	TCACAGAGCCTGTGGCGAAAC
CitERF41	ATGGCTAGGCCACAGCAG	CTAAGTTGAATTGCCACAACAAAGCTC
CitERF42	ATGGAAATTCAATTTCAACAAC	TTACAGAGCATATGAACACAAT
CitERF43	CATGGTACATTCAAAGAAGTTCAGAGG	GATCAATTTTCATTAAGCAATTCTTC
CitERF50	CCCAACTCTCTTCGCCTTAAAAATGAATC	CCGTCAATGCGAATCGGGCAAAGGAGGAG
CitERF51	ATGTCTCCAAAATCCGACATG	CTAGACTTCCACTAAAAAATCG
CitERF52	ATGCCGGAGTCTCGAAAACC	TTAAACGCAAGCAATGTTGAGG
CitERF53	ATGCCTAGCCTTCAAAGG	TTAAAAGCATGTTAAGTCCAG
CitERF55	GGGAGTTCTTTATGAAATTCGC	AGTTTGCTCAATACACTCCC
CitERF57	ATGTGTGGCGGTGCACTTAT	CTAACACAAGAACTGATTGC
CitERF60	ATGGCGCCGAGAGAGAA	TCAGGCGAAATCTTCCGGT
CitERF61	ATGAGGAGGGCGAGAGG	TCAGAGACATAAAGCGGT
CitERF63	CCCCACGTTGATCATCATTTCAATG	TACCGAACATGTGTCAATAC
CitERF68	GCGGCCGCATGCGGCGAGGCAGGA	ACTAGTTCAGAGGCACAATCGAAG
CitERF69	ATGGCTCCGAGAGATAAGCTCA	TCAAGCCACTTCAGGTGGAGG
CitERF70	ATGGCGTCCTCACGTGAG	TCAAAGCCAGAGCGGTGG
CitERF71	ATGAACAGAGAAAATATGTCCGA	TCACCCGGGTCTAAAGGACA
CitERF72	CGCGGTGGCGGCCGCCCTAATGGATTCCTTGTTCTTCC	GGGGGATCCACTAGTTCACCAATTAGCGAGGTCTTG
CitERF73	ATGTTTCCGGCCTCCCAAAT	TTACTCCGATTCCGATCTC
CitERF76	ATGTCCAACATGACAACGTT	CTAGATAATTGAGATGGTTG
CitERF78	GGCATTCGCGCACAAACATGAAAGC	CTAGTTTTACTCTTTACCAGGCC
CitERF80	ATGGAATCATCTTGTCTTC	TCACCAAATAGTAGCAGTAG
CitERF83	AACAGAAGGCCATGGATGAGGAGCC	GCTTCACCCTTTCTTGTTCCTT

CitERF85	CTGCCTACCAAAAATGCAGAAC	CTAGATCAGATGAGAGCATCCG
CitERF89	TTTGAAATGGCAGCTGGTCATC	ATTTAGCAAACGCCCAACTGGCCAAAGC
CitERF92	ATGATTTCTCATCTTCACCATC	TCAATTTACCAATAAGTGCTCG
CitERF93	ATGTGTGAAAGGTTGCAGTT	TCAGTTTACCAATAGCTGGT
CitERF97	GAATCAGACATGTCTGCCATGG	CTACCCATTTGGGTTGTTAC
CitERF99	ATGTCTGCTATGGTTTCAGCCC	CTAATCATCTGGGTGACTTGTA
CitERF102	ATGCAAAGATCCTCAAAGCGAC	TCATGAAGTAAGACCAGTGGCA
AtWRI1	CGCGGTGGCGGCCGCATGAAGAAGCG CTTAACCACTTCC	GGGGGATCCACTAGTGATTATTCAGAACC AACGAACAAG
AtWRI2	CGCGGTGGCGGCCGCGAAGAAGGTTC CGATCATGGC	GGGGGATCCACTAGTTCATTTCTCTTGTG GGAGGTAG
AtWRI3	CGCGGTGGCGGCCGCATGTTCATCGCC GTCGAAGTTTC	GGGGGATCCACTAGTTTAGCAATCATTTA ACTCGCTG
AtWRI4	CGCGGTGGCGGCCGCCTTCTCATTCAC CGGTAATG	GGGGGATCCACTAGTTTAAGGTCCATAAT CAAACTC
Promoter of CitTPS1	GAATATTTAACTTTTTCCGTTC	GTCTGACACAACCATGGCGTCTGG

Table S3. Primers for real-time PCR

Gene name	Forward primers (5' to 3')	Reverse primers (3' to 5')
Citrus actin	CATCCCTCAGCACCTTCC	CCAACCTTAGCACTTCTCC
CsTPS1	ACGACTAGTTCTATACTTCTA	AGTACAGACCAATACGGTACACC

Table S4. Contents of 119 volatile compounds in citrus fruit from different species

No.	Volatile compounds	Mandarin		Sweet Orange		Sour orange	Hybrids		Pomelo		Citron	
		Satsuma	Ponkan	Newhall	Fengjie	Goutoucheng	Huyou	Hongshigan	Yuhuan	Zaoxiang	Lemon	Bagermot
						**Monoterpenes**						
mt1	α-Thujene	0.17cde	0.22cd	0.04f	0.04f	0.11def	0.28bc	0.26c	0.05ef	0.05ef	0.39b	0.86a
mt2	α-Pinene	1.17cde	1.53bcd	0.53e	0.89de	1.19cde	1.45cd	1.88bc	0.71e	1.05de	2.21ab	2.87a
mt3	Camphene	—	—	—	—	0.02c	—	—	0.03b	0.04b	0.09a	0.04b
mt4	Sabinene	—	—	0.55c	0.69b	—	—	1.19a	—	—	—	—
mt5	β-Pinene	0.61e	0.88e	—	—	2.02d	1.15e	—	3.01c	3.98b	8.87a	2.17d
mt6	Myrcene	2.99bc	3.02bc	2.68bcd	3.06bc	2.79bcd	3.22b	3.10bc	26.94a	3.21b	1.38d	1.66cd
mt7	α-Terpinene	—	—	—	—	—	0.24b	—	0.07c	—	0.30b	0.71a
mt8	D-Limonene	59.30bc	62.80abc	60.45bc	65.16ab	57.39c	60.14bc	59.43bc	42.77d	67.31a	29.80e	28.83e
mt9	*E*-ocimene	—	—	0.03de	—	—	0.02de	—	0.06cd	0.10b	0.06c	0.55a
mt10	*Z*-ocimene	0.18cd	0.11d	0.13d	0.1d	0.33c	0.18cd	0.29c	1.64b	1.96a	0.21cd	1.53b
mt11	γ-Terpinene	5.47d	7.19c	0.15f	0.12f	4.12e	8.37b	6.64c	0.29f	0.09f	6.70c	15.54a
mt12	Terpinolene	0.58b	0.62b	0.38c	0.23d	0.37c	0.63b	0.63b	0.13de	0.1e	0.52b	1.31a
mt13	(*4E,6Z*)-allo-Ocimene	0.04de	0.02de	0.04de	0.02de	0.03de	—	0.03de	0.10bc	0.15b	0.06cd	0.54a
mt14	(+)-4-Carene	—	—	—	—	0.02a	—	0.03a	—	—	—	—
mt15	1,3,6-Heptatriene, 2,5,5-trimethyl-	—	—	—	—	0.03a	—	—	—	—	—	—
mt16	Elixene	—	—	—	—	—	—	—	—	—	0.13a	0.08b
						**Sesquiterpenes**						
st1	δ-Elemene	0.61c	0.38cd	—	—	2.46a	1.63b	—	0.75c	0.60c	—	—
st2	α-Cubebene	0.10bc	—	—	—	0.35a	0.14b	0.05cd	0.04cd	0.05cd	—	0.02cd
st3	Ylangene	—	—	—	—	0.04a	0.04a	—	—	—	—	—
st4	Copaene	—	—	—	—	0.84a	—	—	—	—	—	—
st5	(-)-β-Bourbonene	—	—	—	—	—	—	—	—	0.04a	—	—

st6	β-Elemene	0.15a	—	—	—	—	0.09b	0.08bc	—	0.04cd	—	—
st7	α-Gurjunene	—	—	—	—	0.04a	—	—	—	—	—	—
st8	Caryophyllene	—	—	0.64ab	0.60b	—	1.09a	—	0.53b	0.90ab	0.71ab	0.67ab
st9	(+)-Cycloisosativene	—	—	—	—	2.12a	—	—	—	—	—	—
st10	α-Santalene	—	—	—	—	—	—	—	—	—	0.03a	—
st11	β-Cadinene	0.07bc	0.03bc	0.07bc	0.07bc	0.43a	0.44a	0.07bc	0.23ab	0.21b	—	0.05bc
st12	γ-Elemene	0.18c	0.29c	—	—	0.72b	2.03a	—	0.22c	0.22c	—	—
st13	(+)-Aromadendrene	—	—	—	—	—	—	—	—	—	0.03a	0.03a
st14	α-Guaiaene	—	—	—	—	—	0.03a	—	—	—	—	—
st15	α-Bergamotene	—	—	—	—	0.09c	—	—	—	—	0.87a	0.46b
st16	α-Caryophyllene	0.29bc	0.05d	0.07d	0.05d	0.56a	0.41ab	0.03d	0.13cd	0.16cd	0.07d	0.08d
st17	(+)-Epi-bicyclosesquiphellandrene	0.03cd	—	—	—	0.16b	0.21a	—	0.05c	0.05c	—	—
st18	β-Santalene	—	—	—	—	—	—	—	—	—	0.05a	0.04a
st19	β-Farnesene	—	0.04cd	0.02cd	0.03cd	0.02cd	0.37a	0.11b	—	—	0.13b	0.05c
st20	Selina-3,7(11)-diene	—	—	0.02a	—	—	—	—	—	—	—	—
st21	α-Elemene	—	—	—	—	0.17a	0.12b	—	—	—	—	—
st22	Germacrene D	0.38b	0.14b	0.04b	0.04b	3.14a	3.04a	0.05b	0.76b	0.97b	—	0.30b
st23	Selinene	0.07cd	—	0.09bc	0.05cd	—	0.04de	—	0.16a	0.13ab	—	—
st24	β-Panasinsene	—	—	—	—	—	—	—	0.12a	—	—	—
st25	(+)-Valencene	—	—	1.60a	0.948b	—	—	0.04c	0.75b	0.79b	0.29c	—
st26	α-Muurolene	—	—	0.04b	0.03b	0.66a	0.62a	0.02b	0.07b	0.07b	—	—
st27	Germacrene B	—	—	—	—	—	—	0.02c	—	—	0.13a	0.12b
st28	δ-Guaiene	0.07a	—	—	—	0.05ab	0.04b	—	—	—	—	—
st29	*cis*-α-Bisabolene	—	—	—	—	—	—	—	—	—	0.14a	0.10b
st30	γ-Cadinene	0.08b	0.01bc	—	—	0.30a	0.32a	0.01bc	—	—	—	—
st31	(-)-α-Panasinsen	—	—	—	—	—	—	—	0.27a	0.27a	—	—

st32	β-Bisabolene	—	—	—	—	—	—	—	—	—	1.71a	1.10b
st33	α-farnesene	1.41a	0.05c	0.03c	0.04bc	0.10bc	—	0.23bc	0.32b	0.11bc	—	—
st34	β-Himachalene	—	—	—	—	—	—	—	—	—	0.03a	—
st35	(+)-δ-Cadinene	0.39bc	0.07c	0.14c	0.15c	1.56a	0.78b	0.18c	—	0.17c	0.04c	0.09c
st36	β-Sesquiphellandrene	—	—	—	0.02b	—	0.04a	—	—	—	—	—
st37	Cadine-1,4-diene	0.03b	—	—	—	0.10a	0.08a	—	—	—	—	—
						**Monoterpenoids**						
mtd1	*cis*-β-terpineol	0.16c	0.11cd	0.27b	0.14c	0.04d	0.05d	0.09cd	0.08cd	0.09cd	0.06d	0.49a
mtd2	*cis*-p-Menth-2-en-1-ol	0.32b	0.01c	0.05d	0.03d	0.05d	0.04d	0.11cd	0.03d	0.03d	0.12cd	1.26a
mtd3	Linalool	7.33a	5.55abc	6.29ab	5.06bc	7.29a	1.13e	3.98cd	2.02de	2.45de	1.04e	1.53e
mtd4	1,3,8-p-Menthatriene	0.06d	0.02de	—	0.02de	—	0.02de	0.04bc	0.01ef	0.03cd	0.03cd	0.05ab
mtd5	*trans*-p-Mentha-2,8-dien-1-ol	0.14b	0.05d	0.08d	0.06d	0.04d	0.08cd	0.13b	0.13bc	0.24a	0.04d	0.08cd
mtd6	*Z*-Limonene oxide	—	—	0.05b	0.03c	—	—	0.08a	—	0.06b	—	—
mtd7	*E*-Limonene oxide	0.22a	0.15bc	0.22a	0.14c	0.02d	0.21ab	0.14c	0.11c	—	—	0.03
mtd8	*cis*-p-Mentha-2,8-dien-1-ol	—	—	—	—	0.02de	0.06bc	0.12a	—	0.08b	—	0.04cd
mtd9	Camphor	0.02cd	0.02c	0.01de	0.02cd	0.03c	—	0.02cd	—	—	0.11b	0.14a
mtd10	Menthol	—	0.04a	—	—	—	—	—	—	—	—	—
mtd11	Isopulegol	—	—	—	0.02a	—	—	—	—	—	—	—
mtd12	(R)-(+)-Citronellal	0.72bc	1.90a	0.55cd	0.64c	0.92b	0.46cde	0.32efd	0.20ef	0.15f	0.25ef	0.34efd
mtd13	Borneol	0.03c	—	—	—	—	—	—	—	—	0.13a	0.04b
mtd14	4-terpineol	0.83b	0.42cd	0.27de	0.21de	0.25de	0.53c	0.42cd	0.09e	0.17e	1.06b	1.98a
mtd15	α-terpineol	3.83b	2.02cd	2.28c	1.86cde	1.84cde	1.15ef	1.75cde	0.78f	1.54de	4.32b	7.60a
mtd16	(*E*)-p-Menth-8-en-2-one	—	—	—	—	—	—	0.05a	—	—	—	—
mtd17	*Z*-Carveol	0.41a	0.08de	0.30b	0.15cd	—	—	0.18c	—	0.40e	0.11cd	—
mtd18	*Z*-Geraniol	0.41def	0.18ef	0.65bcde	0.35ef	0.05f	0.13f	1.23a	0.91abcd	0.95abc	0.55cdef	1.08ab
mtd19	Citronellol	0.48b	0.98a	0.28c	0.20cd	0.09de	0.17cde	—	0.11cde	0.10cde	—	0.12cde

mtd20	Neral	0.61ef	0.54ef	4.44c	3.95c	1.60de	0.47ef	1.29de	4.24c	2.17d	11.99a	9.05b
mtd21	Piperitone	0.04cd	0.05c	0.02de	0.03cd	0.04cd	0.02de	—	0.03cd	0	0.09b	0.15a
mtd22	*E*-Geraniol	0.13c	0.12c	0.31bc	0.26bc	0.08c	0.09c	0.07c	0.69b	0.28bc	0.51bc	1.24a
mtd23	(-)-Perillaldehyde	2.05a	0.79b	0.46cd	0.60bc	0.52bcd	0.23ed	0.63bc	—	0.35cd	—	—
mtd24	*E*-Geranial	0.43f	0.67ef	6.13c	5.37c	2.22de	0.42f	1.26ef	6.16c	2.84d	19.07a	13.35b
mtd25	Bornyl acetate	—	—	—	—	0.04a	0.03b	—	—	—	—	—
mtd26	Indole	—	—	—	—	—	0.09b	—	0.11b	0.18a	—	—
mtd27	p-Mentha-1,8-dien-7-ol	—	—	—	—	—	—	—	—	0.07a	—	—
mtd28	Carvacrol	—	—	—	—	—	0.05a	—	—	—	0.03b	—
mtd29	Thymol	—	—	—	—	—	0.14b	0.25a	—	—	0.05c	0.15b
mtd30	Terpinyl acetate	—	—	—	—	—	—	0.07a	—	—	—	—
mtd31	Citronellyl acetate	0.15b	0.19ab	0.06d	0.04d	—	0.21a	0.05d	0.06d	—	0.11c	—
mtd32	Neryl acetate	0.60c	0.23efg	0.21efg	0.12fg	—	1.37b	0.46cd	0.30dfe	0.43cde	2.36a	0.08fg
mtd33	Geranic acid methyl ester	—	—	—	—	—	—	—	—	—	0.04a	—
mtd34	m-Menthane	—	—	—	—	—	—	—	—	—	0.03a	—
mtd35	Geranyl acetate	—	—	—	—	—	—	—	—	—	1.21a	0.08b
mtd36	β-Ionone	—	—	—	—	—	—	—	—	—	—	0.04a
						**Sesquiterpenoids**						
std1	Safranal	—	—	0.02a	0.02a	—	—	—	—	—	—	—
std2	Elemol	0.07bc	—	0.03cd	0.02d	0.09b	0.14a	0.01d	—	—	—	—
std3	*E*-Nerolidol	—	—	—	—	0.09b	—	—	0.04c	0.15a	—	—
std4	Caryophyllene oxide	—	—	0.05a	0.05a	—	0.06a	—	—	—	—	—
std5	.tau.-Muurolol	—	—	—	—	—	0.02a	—	—	—	—	—
std6	α-Bisabolol	—	—	—	—	—	—	—	—	—	0.06a	0.04b
std7	Farnesol	—	—	—	—	—	—	—	0.11a	0.08a	—	—
std8	*E*,*E*-Farnesal	—	—	—	—	—	—	—	0.02a	—	—	0.02a

std9	α-Sinensal	—	—	0.02a	0.03a	—	—	—	—	—	—	—
std10	Nootkatone	—	—	0.02b	0.01b	0.05b	0.36a	—	0.47a	0.36a	—	—
**Aliphatic Aldehydes and Alcohols**												
aad1	Hexanal	—	—	—	—	—	—	—	0.38b	0.65a	—	—
aad2	(E)-2-Hexenal	1.02ab	0.16ef	0.54cde	0.40def	0.06f	1.12a	0.22ef	0.69bcd	0.87abc	0.08f	0.05f
aad3	Heptanal	—	—	—	—	—	—	0.03a	—	—	0.02b	0c
aad4	Octanal	2.94abc	2.24bcd	4.74a	3.37ab	1.07d	1.38cd	4.71a	1.14cd	1.59bcd	0.58d	0.88d
aah	1-Octanol	0.58a	0.39abc	0.51ab	0.65a	—	0.17bcd	0.57a	0.04cd	0.03cd	—	—
aad5	Nonanal	0.47bc	0.38c	0.45bc	0.47bc	0.12d	0.42c	0.66a	0.07d	0.06d	0.56ab	0.11d
aad6	(E)-2-Nonenal	0.02a	0.02a	—	—	—	—	—	—	—	—	—
aad7	Decanal	1.39c	2.94b	2.78b	2.97b	0.73cde	1.25cd	4.65a	0.43e	0.56de	0.25e	0.09e
aad8	2-Decenal	—	0.41a	—	—	—	—	0.11b	—	—	—	—
aad9	2,4-Decadienal	—	0.28a	—	—	—	—	—	—	—	—	—
aad10	Dodecanal	0.12bc	0.15b	0.12bc	0.10c	0.04d	0.15b	0.29a	0.03d	0.04d	0.16b	0.07cd
aad11	(E,E)-2,4-Decadienal	0.07bc	0.54a	0.05bcd	0.09bc	0.05bcd	0.05bcd	0.12b	0.04cd	0.06bcd	—	—
aad12	Tridecanal	—	0.58b	—	—	—	—	1.11a	—	—	—	—
aad13	Tetradecanal	—	0.03a	—	—	—	—	0.02a	—	—	—	—
**Aliphatic Esters**												
ae1	Acetic acid, nonyl ester	—	—	—	—	—	—	—	—	—	0.04a	—
ae2	Acetic acid, heptyl ester	—	—	—	—	—	0.03a	0.03a	—	—	—	—
ae3	Acetic acid, octyl ester	—	0.07de	—	0.11cd	0.16c	0.41a	0.26b	—	—	—	—
ae4	Hexanoic acid, hexyl ester	—	—	—	—	—	—	—	0.21a	0.09b	—	—
ae5	Acetic acid, decyl ester	0.02c	0.04ab	—	0.02cd	0.05a	0.05ab	0.05a	—	0.03bc	—	—
ae6	Octanoic acid, hexyl ester	—	—	—	—	—	—	—	0.05a	—	—	—

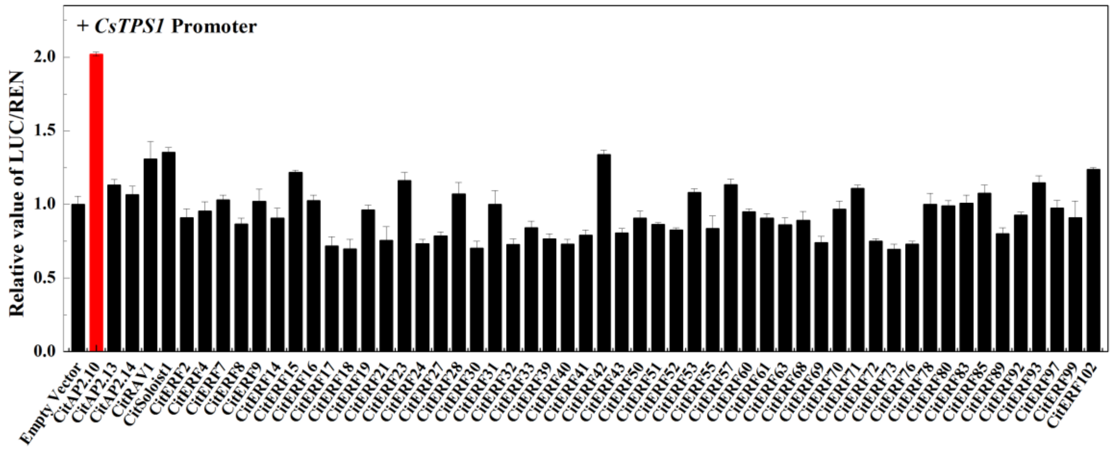

Figure S1. In vivo interaction of ethylene responsive factors and the promoter of *CsTPS1*.

Samples were infiltrated into Nicotiana benthamiana leaves. For the TF-promoter interaction, three independent experiments were performed (at least four replicates in each experiment). Firefly luciferase and renilla luciferase were assayed 3 days after infiltration. The ratio of LUC/REN of the empty vector (SK) plus promoter was used as calibrator (set as 1). Error bars indicate SEs from four biological replicates.

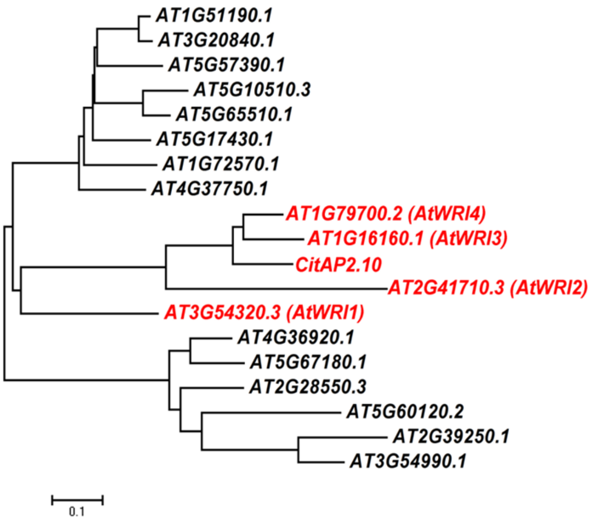

Figure S2. Phylogenetic analysis of *CitAP2.10* and Arabidopsis *AP2* genes. The amino acid sequences were obtained from TAIR (The Arabidopsis Information Resource).
